# Preparation of Steel-Slag-Based Hydrotalcite and Its Adsorption Properties on Cl^−^ and SO_4_^2−^

**DOI:** 10.3390/ma16237402

**Published:** 2023-11-28

**Authors:** Zebo Dong, Bei Huang, Tao Zhang, Na Liu, Zhongyang Mao

**Affiliations:** 1 College of Materials Science and Engineering, Nanjing Tech University, Nanjing 211800, China; 202161103094@njtech.edu.cn (Z.D.); 201961203162@njtech.edu.cn (T.Z.); 202161103117@njtech.edu.cn (N.L.); mzy@njtech.edu.cn (Z.M.); 2 State Key Laboratory of Materials-Oriented Chemical Engineering, Nanjing 211800, China

**Keywords:** steel slag, hydrotalcite, hydrothermal synthesis, adsorption performance

## Abstract

Large amounts of chloride ions (Cl^−^) and sulfate ions (SO_4_^2−^) are present in salt-washing wastewater, making it unsuitable for direct release. Adsorption can be used to eliminate Cl^−^ and SO_4_^2−^ from salt-washing wastewater, and hydrotalcite is an excellent adsorbent with high adsorption properties for these ions because of a layered bimetallic hydroxide structure. The selective extraction of various metals, such as calcium, magnesium, aluminum, and iron, from steel slag via acid leaching facilitates the utilization of steel slag in the preparation of hydrotalcite. In this study, the leaching mechanism of metal in steel slag was investigated using steel slag as a raw material and acetic acid as the reaction medium. The study obtained the optimal leaching mechanism for preparing hydrotalcite. Hydrotalcite was synthesized from the steel slag leaching solution by hydrothermal synthesis, and its structure was characterized. The adsorption performance of Cl^−^ and SO_4_^2−^ in salt-washing wastewater was investigated by solution adsorption experiments. The removal rates of Cl^−^ and SO_4_^2−^ in salt-washing wastewater reached 12.8% and 38.0%, respectively. After multiple adsorption cycles, the removal rates increased to 98.0% for Cl^−^ and 96.4% for SO_4_^2−^.

## 1. Introduction

Soil salinization in the western region of China not only impedes the sustainable development of the agricultural economy but also presents significant resource issues. The process of “irrigation and salt washing” has been demonstrated to be a highly effective method for enhancing saline soils [1,2]. However, the salt-washing wastewater created from this technique has substantial amounts of Cl^−^ and SO_4_^2−^, which would be an environmental burden if discharged directly [3]. Adsorption is a common method used for removing Cl^−^ and SO_4_^2−^ from wastewater generated during salt washing. Hydrotalcite, an adsorbent material that has interlayer anion exchangeability [4], has been widely used to remove harmful impurities such as phosphate [5,6], lead [7], and arsenic [8] from water. It is also effective in adsorbing Cl^−^ and SO_4_^2−^ in salt-washing wastewater [9]. There are generally two types of raw materials used in producing hydrotalcite: pure chemicals and solid waste.

Steel slag, a solid waste produced by the iron and steel industry, is currently a focal point of research [10]. Steel slag contains iron, CaO, and MgO, which can be utilized as sintering materials [11]. Additionally, steel slag has been demonstrated to be environmentally friendly and possesses cementing properties when used as a subgrade material [12,13]. Steel slag can also be applied to prepare microcrystalline glass. Zhao Guizhou et al. prepared microcrystalline glass with varying alkalinity using steel slag, via a one-step sintering method [14]. They also discovered that combining steel slag with coal gangue resulted in microcrystalline glass with a heavy metal ion curing efficiency exceeding 90% [15]. In addition, steel slag has potential for use in the preparation of silicate cement and sulphoaluminate cement [16,17,18,19]. Yang Kaimin et al. showed that using steel slag as a substitute for iron raw materials did not change the main mineral composition of the clinker and resulted in a more comprehensive development of alite [20]. Deqiang Zhao et al. successfully prepared multiphase cement clinker from granular steel slag using multiphase sintering technology [21,22]. Since calcium silicate in steel slag has cementitious activity, it can be used as a cement admixture in production [23,24,25]. Nurul Hidayah Roslan and colleagues conducted research on replacing 20% of the cement in concrete with steel slag, which resulted in higher compressive strength at a later stage of curing compared to the control group [26]. Extracting metal elements from steel slag through acid leaching provides an opportunity for comprehensive utilization. This process leaches metal oxides, such as calcium, magnesium, aluminum, and iron, from the steel slag. These metal oxides can then be utilized for preparing hydrotalcite. Steel-slag-derived hydrotalcite significantly enhances the flame-retardant properties of materials [27]; Guanhao Liu et al. employed steel slag after nitric acid acidolysis to prepare roasted hydrotalcite by the co-precipitation method, which yields high catalytic activity and good stability [28]. It is worth noting that most acidolysis methods for steel slag utilize strong acids such as nitric acid and sulfuric acid [27,28,29]. However, properly treating these strong acid waste products is essential for preventing adverse environmental impacts.

Currently, there are only a few studies that concentrate on producing hydrotalcite from steel slag. This paper employs acetic acid to acidify the steel slag to investigate the leaching mechanism of metal elements in it under various process conditions. The hydrothermal process is used to prepare hydrotalcite from the leach solution of the steel slag. The study examines the adsorption effectiveness of hydrotalcite prepared for Cl^−^ and SO_4_^2−^ found in salt-washing wastewater. The outcomes will help reduce costs linked with hydrotalcite production and enhance the resource utilization of valuable metals within steel slag, thus promoting sustainable and efficient use.

## 2. Materials and Methods

### 2.1. Raw Materials

**Figure 1:**
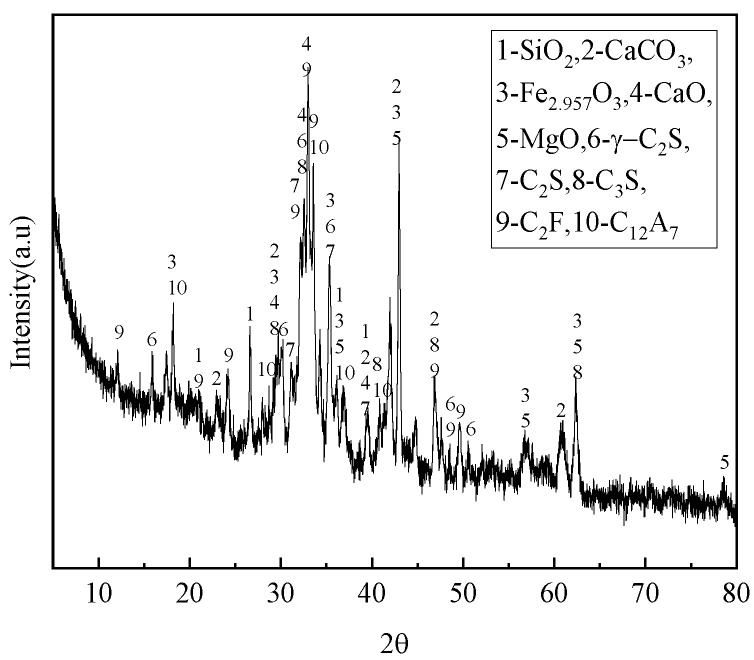
XRD pattern of steel slag.

**Figure 2:**
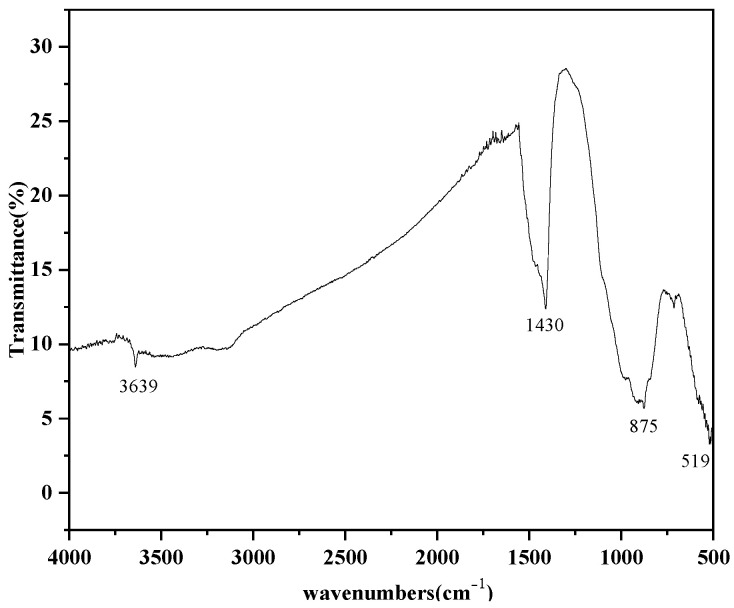
The FTIR patterns of steel slag.

The steel slag was provided by Oriental Runan Group Co. (Changzhou, China), processed by ball milling, and sieved through 20 mesh. Its chemical and mineral composition are shown in Table 1 and Figure 1. Steel slag mainly contains silica, calcium carbonate, RO phase (oxides of magnesium, iron, etc.), olivine, tricalcium silicate, dicalcium silicate, C_2_F, C_12_A_7_, and so on. Figure 2 presents the FTIR pattern of the steel slag.

The salted soil originating from Xinjiang was dried naturally and sieved through a 1 mm nylon sieve. Then, 20 g of salted soil was weighed into a conical flask, 100 mL of deionized water was added, and it was placed in a constant-temperature water bath oscillator and oscillated at a constant temperature of 25 °C for 2 min, and the salt washing wastewater was filtered immediately after the end of oscillation. The concentrations of Cl^−^ and SO_4_^2−^ in the salt-washing wastewater were 12,486 mg/L and 6602 mg/L, respectively. The chemical reagents used in this paper are acetic acid (CH_3_COOH), anhydrous ethanol, sodium hydroxide, and deionized water, all of which are analytically pure. Acetic acid was obtained from Sinopharm Group Chemical Reagent Co. (Shanghai, China) and NaOH was obtained from Xilong Science Co., Ltd. (Shantou, China); anhydrous ethanol was purchased from Wuxi Yasheng Chemical Co. (Wuxi, China).

### 2.2. Acidolysis of Steel Slag

Initially, the three-necked flask is prepared for the experiment by adding the acetic acid solution and inserting the thermometer. Once the thermometer reaches the desired temperature, steel slag is introduced to the flask. Stirring is ceased after a specific amount of time, and the resulting mixture is filtered by a pump to acquire the leaching solution of the steel slag.

The effect of acetic acid concentration on the selective leaching of target ions from steel slag was considered. Five different acetic acid concentrations of 3 mol/L, 4 mol/L, 5 mol/L, 6 mol/L, and 7 mol/L were selected during the experiment. To investigate the effect of liquid–solid ratio on ion leaching, five different liquid–solid ratios of 10 mL/g, 15 mL/g, 20 mL/g, 25 mL/g, and 30 mL/g were selected for the experiment. To investigate the effect of reaction time and reaction temperature on ion leaching, three different temperatures of 70 °C, 80 °C, and 90 °C were selected, respectively.

### 2.3. Preparation of Hydrotalcite

Hydrotalcite was synthesized hydrothermally using steel slag leach solution as the primary material. A set amount of said solution was added to a beaker, with a thermometer utilized to measure the temperature throughout the reaction. The beaker was placed in a magnetically stirred water bath for mixing, and the reaction temperature was maintained at <40 °C throughout the bath. A pH meter (Shanghai Lei Magnetics Co., Shanghai, China, PHS-25) was inserted for pH measurement during the reaction; sodium hydroxide solution was added while stirring to adjust the pH to the desired value. The slurry obtained from the reaction is transferred to the hydrothermal reactor. Then, the hydrothermal reactor is put into the oven to be heated at a specific temperature for a particular time. After the reaction, the hydrothermal reactor was removed and cooled to room temperature, and the supernatant was poured off. The remaining material was washed numerous times with deionized water and anhydrous ethanol, and neutralized through centrifugation and filtration. The filter residue was dried to a constant weight in an oven at 80 °C, and then milled to obtain the Ca-Mg-Al-Fe hydrotalcite.

In order to investigate the effect of pH on the formation of phases, the pH was adjusted to 9, 10, 11, and 12 with sodium hydroxide. In order to investigate the effect of reaction temperature on the formation of phases in the hydrothermal reaction, four different reaction temperatures were chosen: 60 °C, 90 °C, 120 °C, and 150 °C. Four different reaction times, 12 h, 16 h, 20 h, and 24 h, were chosen to investigate the effect of reaction time on the formation of phases in the hydrothermal reaction.

### 2.4. Adsorption of Cl^−^ and SO_4_^2−^ from Salt-Washing Wastewater by Hydrotalcite

Six 50 mL portions of salt-washing wastewater were taken, and 3 g of hydrotalcite was added to the solution. The solutions were placed on a thermostatic oscillator at a temperature of 25 °C and a speed of 150 rpm for 6 h. A portion of the solution was removed every 1 h filtered, and the concentrations of Cl^−^ and SO_4_^2−^ in the remaining solution were measured. The above experiment was repeated several times to measure the concentrations of Cl^−^ and SO_4_^2−^ in the remaining solution.

The adsorption of Cl^−^ and SO_4_^2−^ in salt-washing wastewater is calculated as in Equation (1):

(1) $$q=\frac{(c_{0}-c_{r})\times V}{m}$$

where:

q: Adsorption amount of hydrotalcite (mg/g);
C_0_: Initial concentration of Cl^−^ and SO_4_^2−^ (mg/L);
C_r_: Ion concentration (mg/L) at the end of adsorption;
V: Volume of solution (L);
m: Amount of hydrotalcite added (g).

### 2.5. Testing Methods

The steel slag leachate samples were diluted 1000 times and analyzed by inductively coupled plasma optical emission spectroscopy (ICP-OES, Perkin EImer, Waltham, MA, USA, Optima 7000DV) by analyzing the content of Ca^2+^, Mg^2+^, Al^3+^, Fe^3+^, and Si^4+^. Ion chromatography ion chromatograph (IC, Metrohm, Herisau, Switzerland, ECO) was used to measure the concentrations of Cl^−^ and SO_4_^2−^ in the salt-washing wastewater before and after hydrotalcite adsorption.

The prepared hydrotalcite was ground to a fine powder using an agate mortar, and the hydrotalcite was examined using X-ray diffraction (XRD, Rigaku, Tokyo, Japan, Smart Lab). The test conditions were the copper target, 30 Kv, 15 mA, scanning angle 10–90°, and scanning rate 10°/min.

The prepared hydrotalcite powders were analyzed using field emission scanning electron microscopy (FESEM, JEOL, Tokyo, Japan, JSM-6701F) and transmission electron microscopy (TEM, FEI, Hillsboro, OR, USA, FEI TF20) to investigate the morphology of hydrotalcite further.

The prepared hydrotalcite samples were analyzed using Fourier-transform infrared spectroscopy (FT-IR, Thermo Fisher Scientific, Waltham, MA, USA, Nicolet is50) to determine the type of bonds contained in the hydrotalcite structure, with a 400–4000 cm^−1^ test wave number.

Steel slag, prepared hydrotalcite samples, and adsorbed hydrotalcite samples were tested using a Surface Area and Porosimetry Analyzer (Gold APP, Beijing, China, V-Sorb 1800) to compare the size of the particular surface area of the hydrotalcite samples and to analyze the changes in their adsorption capacity.

## 3. Results

### 3.1. Determination of Steel Slag Acidolysis System

#### 3.1.1. The Effect of Acetic Acid Concentration

**Figure 3:**
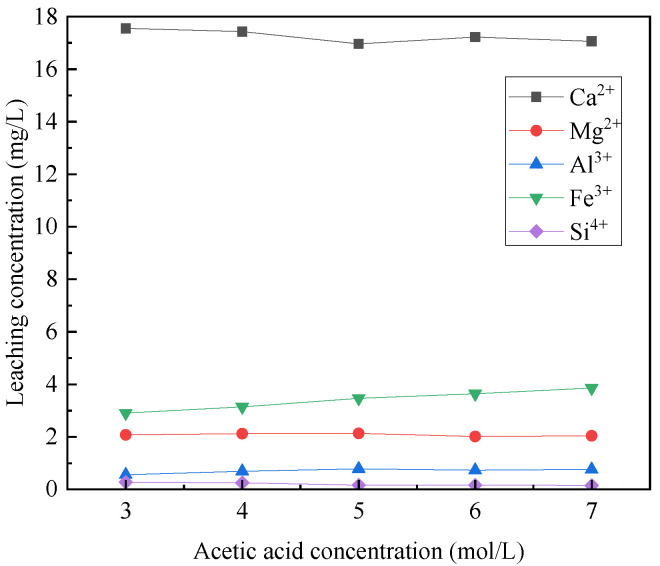
Effect of acetic acid concentration on selective leaching of steel slag.

Figure 3 illustrates the impact of acetic acid concentration on the selective leaching of Ca^2+^, Mg^2+^, Al^3+^, Fe^3+^, and Si^4+^ in steel slag. The results indicate that the concentrations of Ca^2+^ and Mg^2+^ in the leaching solution remain relatively constant, while the concentrations of Al^3+^ and Fe^3+^ gradually increase with the acetic acid concentration increases. Conversely, the concentration of Si^4+^ decreases as the acetic acid concentration increases. Consequently, it can be inferred that the concentration of acetic acid minimally impacts the selective leaching of Ca^2+^ and Mg^2+^ from the steel slag. However, it significantly affects the selective leaching of Al^3+^, Fe^3+^, and Si^4+^. Additionally, it is worth noting that silicon in the steel slag tends to be leached as colloidal particles [30]. As the acetic acid concentration increases, the content of silicate gel may also rise, potentially hindering effective filtration. Therefore, it is crucial to strike a balance between attaining higher levels of Ca^2+^, Mg^2+^, Al^3+^, and Fe^3+^ in the leaching solution and decreasing the concentration of Si^4+^. Consequently, a 5 mol/L acetic acid solution was opted for, in order to accomplish these objectives.

#### 3.1.2. The Effect of Liquid–Solid Ratio

**Figure 4:**
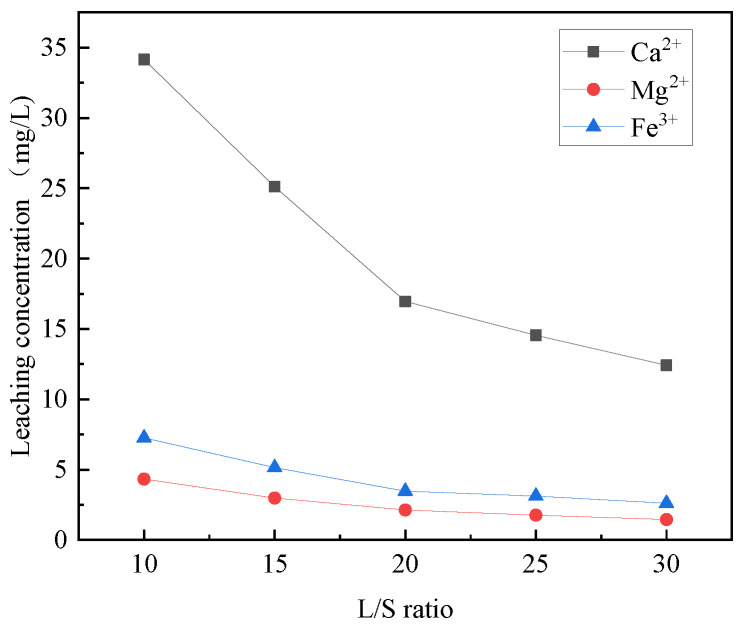
Effect of liquid–solid ratio on selective leaching of Ca^2+^, Mg^2+^, and Fe^3+^.

**Figure 5:**
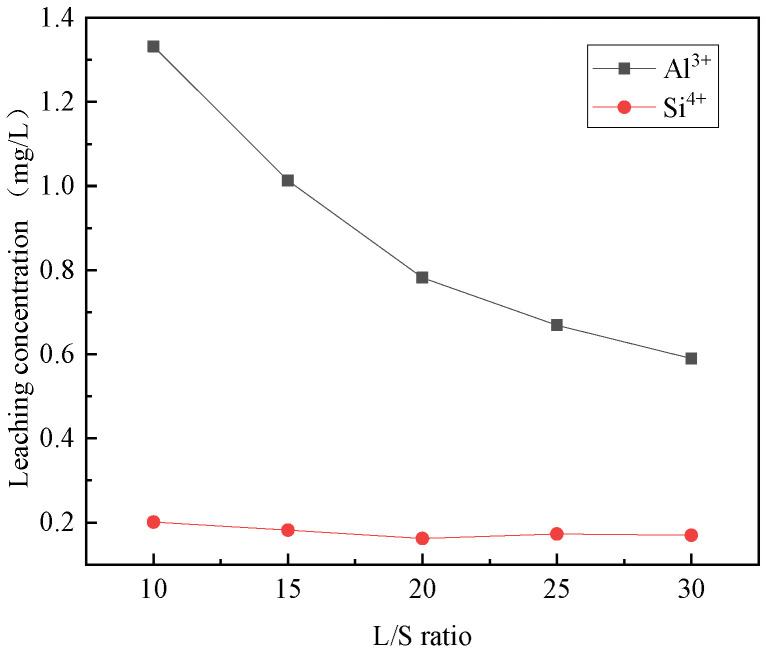
Effect of liquid–solid ratio on the selective leaching of Al^3+^ and Si^4+^.

The effects of different liquid–solid ratios on the selective leaching of Ca^2+^, Mg^2+^, Al^3+^, Fe^3+^, and Si^4+^ from steel slag are shown in Figure 4 and Figure 5. Notably, the liquid–solid ratio plays a significant role in the selective leaching of these ions. Observing the figures, it is evident that the concentration of Ca^2+^, Mg^2+^, Al^3+^, Fe^3+^, and Si^4+^ in the leaching solution decreases with the increase of the liquid–solid ratio. The leach solution exhibits a higher concentration and decrease rate of calcium ions compared to other ions, which have a relatively lower concentration and slower decrease rate. The ratio of trivalent metal cations in the leaching solution to the sum of divalent and trivalent metal cations was calculated. The liquid–solid ratios of 10:1, 15:1, 20:1, 25:1, and 30:1 correspond to 0.148, 0.147, 0.151, 0.155, and 0.156, respectively. None of the ratios are between 0.2–0.33 [31], so it is necessary to choose the closer ones. It is also required to satisfy the relatively high content of Ca^2+^, Mg^2+^, Al^3+^, and Fe^3+^ in the leaching solution and the relatively low content of Ca^2+^, Mg^2+^, Al^3+^, Fe^3+^, and other ions in the residual solution after the preparation of hydrotalcite. Therefore, the liquid–solid ratio of 25:1 was selected.

#### 3.1.3. The Effect of Reaction Temperature

**Figure 6:**
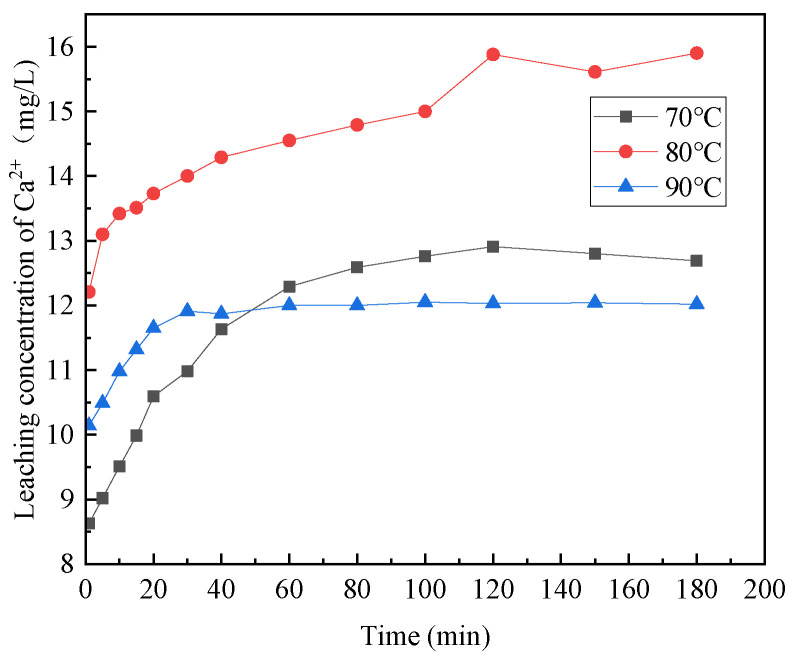
Effect of reaction temperature on the selective leaching of Ca^2+^.

**Figure 7:**
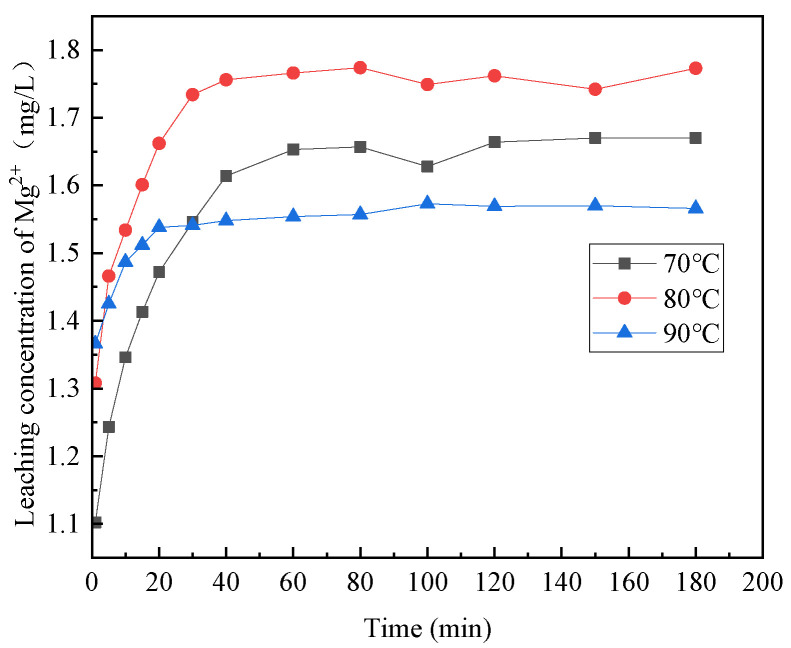
Effect of reaction temperature on the selective leaching of Mg^2+^.

**Figure 8:**
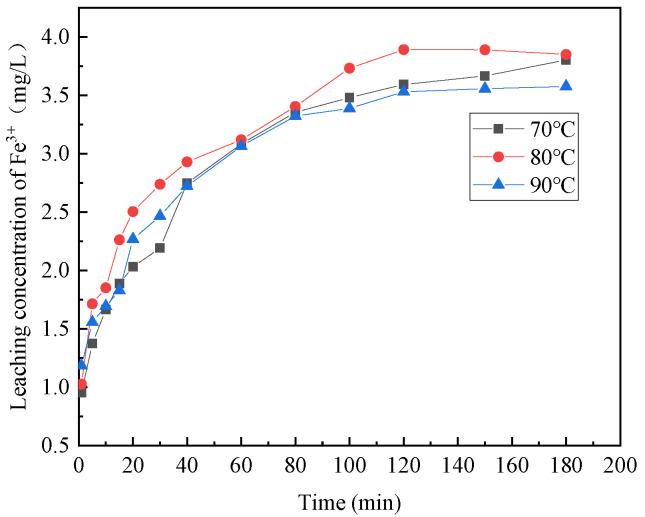
Effect of reaction temperature on the selective leaching of Fe^3+^.

**Figure 9:**
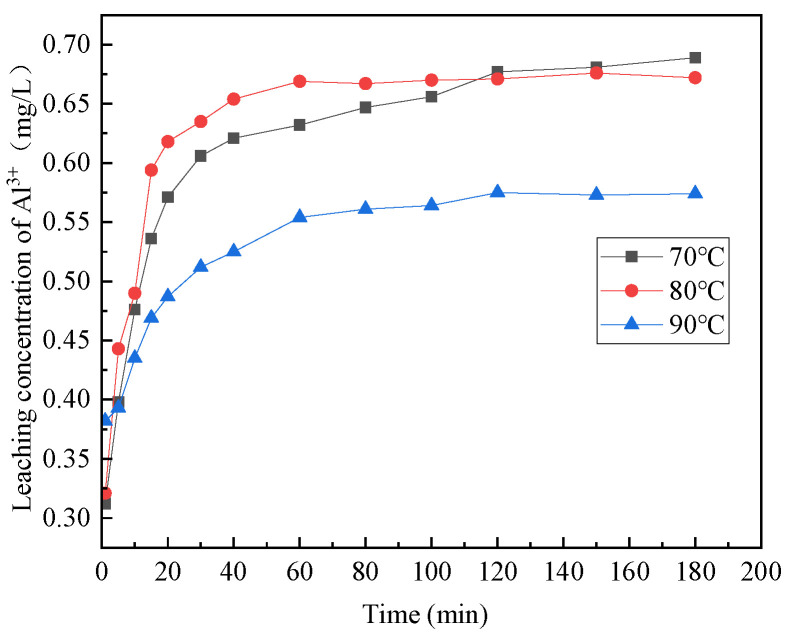
Effect of reaction temperature on the selective leaching of Al^3+^.

**Figure 10:**
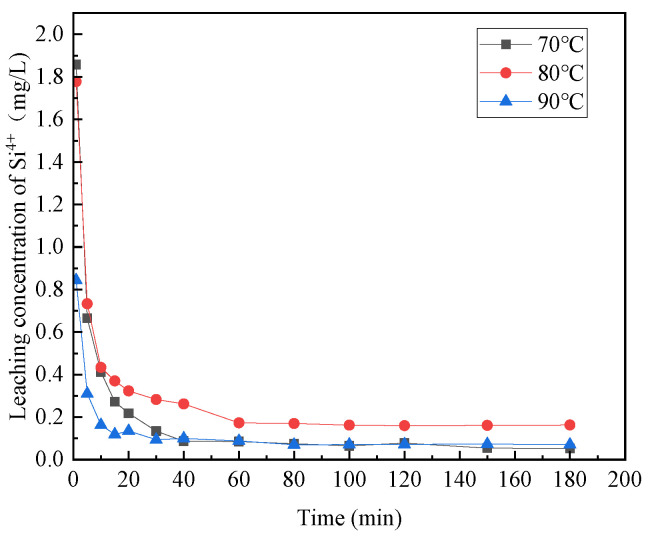
Effect of reaction temperature on the selective leaching of Si^4+^.

Figure 6, Figure 7, Figure 8, Figure 9 and Figure 10 provide insights into the influence of different reaction temperatures and reaction times on the selective leaching of Ca^2+^, Mg^2+^, Al^3+^, Fe^3+^, and Si^4+^ from steel slag, respectively. Examining the figures, the concentration of Ca^2+^, Mg^2+^, Al^3+^, and Fe^3+^ in the leaching solution increased continuously at first, before reaching a steady state, as the acidolysis of steel slag progressed. Conversely, the concentration of Si^4+^ underwent a decrease and then remained constant. The leaching of Ca^2+^ was completed within 120 min, whereas Mg^2+^ and Al^3+^ reached completion within 60 min. Fe^3+^ was leached rapidly in the initial stage of the reaction but slowly in the later stage. The overall leaching rate was relatively slow, and the leaching time was extended, taking about 180 min at 70 °C and 120 min at 80 °C and 90 °C. The Si^4+^ concentration in the leaching solution rapidly decreased in the first 20 min of the reaction and then slowly decreased after 20 min until the end. The figures further indicate that, with the increase in reaction temperature, the concentration of Ca^2+^, Mg^2+^, Al^3+^, Fe^3+^, and Si^4+^ in the leachate reached an extreme value, initially increased and then decreased. The leach solution at 80 °C had the highest concentration of Ca^2+^, Mg^2+^, Al^3+^, Fe^3+^ and Si^4+^ at 15.88 mg/L, 1.762 mg/L, 0.671 mg/L, and 3.891 mg/L, respectively.

Combining the above analyses, the highest concentration of ions was leached at the temperature of 80 °C, so 80 °C was chosen for the subsequent experiments. With the increase of reaction time, Ca^2+^, Mg^2+^, Al^3+^, and Fe^3+^ leaching gradually increased, and Si^4+^ leaching gradually decreased until the ion concentration in the leach solution tends to be the constant at 2 h, so the reaction time was chosen as 2 h for the subsequent experiments.

Therefore, the optimal process conditions for the acidolysis of steel slag were an acetic acid concentration of 5 mol/L, liquid–solid ratio of 25:1, reaction temperature of 80 °C, and reaction time of 2 h. At this time, the concentrations of Ca^2+^, Mg^2+^, Al^3+^, and Fe^3+^ in the leach solution were 15.88 mg/L, 1.762 mg/L, 0.671 mg/L, and 3.891 mg/L, respectively. The ratio of trivalent metal cations in the leaching solution to the sum of divalent and trivalent metal cations was 0.167, and the relatively high content of metal cations in the leaching solution allowed the preparation of more hydrotalcite.

### 3.2. Preparation of Steel-Slag-Based Hydrotalcite

#### 3.2.1. The Effect of pH

**Figure 11:**
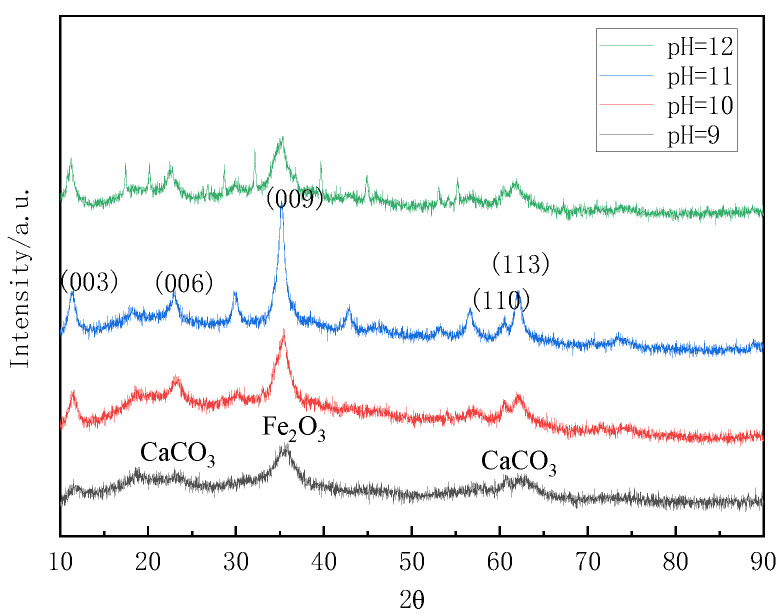
XRD plots of hydrotalcite under different pH conditions.

The experiments utilized the aforementioned steel slag leach solution as the raw material, with pH values adjusted to 9, 10, 11, and 12, a temperature of 120 °C, and a duration of 16 h. Figure 11 showed the XRD patterns of the samples under different pH conditions. By comparing these patterns with the XRD patterns of standard magnesium–aluminum hydrotalcite, it was observed that the diffraction peaks of the samples indicated the presence of CaCO_3_ and Fe_2_O_3_ with no evidence of hydrotalcite formation at pH 9. Conversely, when the pH values were 10, 11, and 12, the diffraction peaks of the hydrothermal reaction product aligned with those of the standard magnesium–aluminum hydrotalcite. The characteristic peaks of crystal surfaces (003), (006), and (009) were present, confirming the formation of hydrotalcite in the product. However, the intensity of the diffraction peaks for the prepared sample was lower compared to those of the standard Mg-Al hydrotalcite, indicating a lower degree of crystal growth along the crystal surfaces. Consequently, the crystallinity of the prepared sample was found to be inferior to that of the standard Mg-Al hydrotalcite.

The XRD test data of samples under different pH conditions is illustrated in Table 2, which lists the corresponding angles and d values of the samples synthesized at pH 10, 11, and 12 on the (003), (006), and (009) crystal planes, as well as the half-peak widths on the (003) plane. It can be seen that the relationship d_(003)_ = 2d_(006)_ = 3d_(009)_ was satisfied at pH 10 and 11, indicating that the hydrotalcite samples had a more regular lamellar structure [32]. Moreover, the half-peak width of 0.0119 rad on the (003) crystalline plane at pH 11 was smaller than that of 0.0142 rad at pH 10, which indicated that the diffraction peaks on the (003) crystalline plane of the sample were sharp and narrow. Thus, the crystallinity was better at pH 11. Therefore, the pH value of 11 was chosen for the subsequent study of the process conditions.

**Figure 12:**
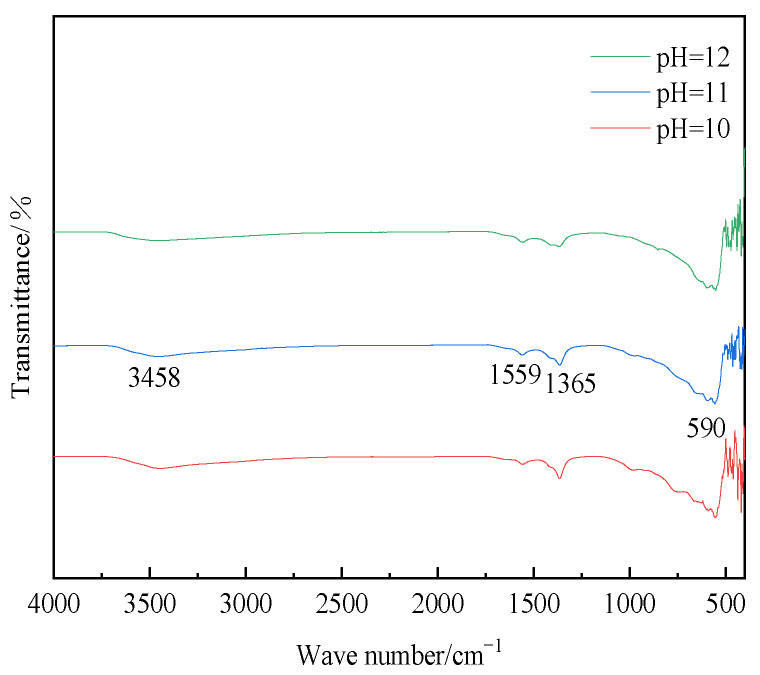
FT-IR plots of hydrotalcite under different pH conditions.

Figure 12 depicted the FT-IR spectra of hydrotalcite at various pH values. As depicted in the figure, the samples prepared at pH values of 10, 11, and 12 exhibited distinct peaks at 3458 cm^−1^, 1559 cm^−1^, 1365 cm^−1^, and 590 cm^−1^. The peak at 3458 cm^−1^ corresponded to the stretching vibrations of H-O-H on the laminar plate and the stretching vibrations of hydroxide bonding in water molecules within the interlayer. The peak at 1559 cm^−1^ represented the bending vibrations of the water of crystallization, while the peak at 1365 cm^−1^ signified the C-O stretching vibrations in CH_3_COO^−^. Additionally, the vibrational absorption peak at 590 cm^−1^ indicated the presence of metal–oxygen bonding. These findings served as further evidence of hydrotalcite synthesis.

**Figure 13:**
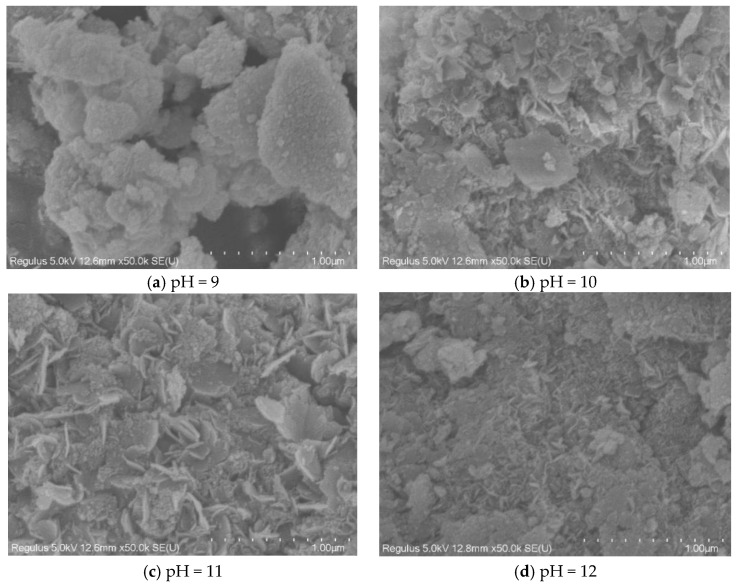
SEM images of hydrotalcite under different pH conditions.

The SEM images of hydrotalcite at different pH values are given in Figure 13, from which it can be seen that hydrotalcite with hexagonal lamellar structure was formed at pH 10, 11, and 12, with a good dispersion of hydrotalcite crystals, uniform surface of the particles, and significant angularity, indicating that the synthesized hydrotalcite had an excellent homogeneity and regularity. The flake structure of hydrotalcite at pH 11 is the most obvious and the most regular, indicating that pH 11 is the optimal condition.

#### 3.2.2. The Effect of Reaction Temperature

**Figure 14:**
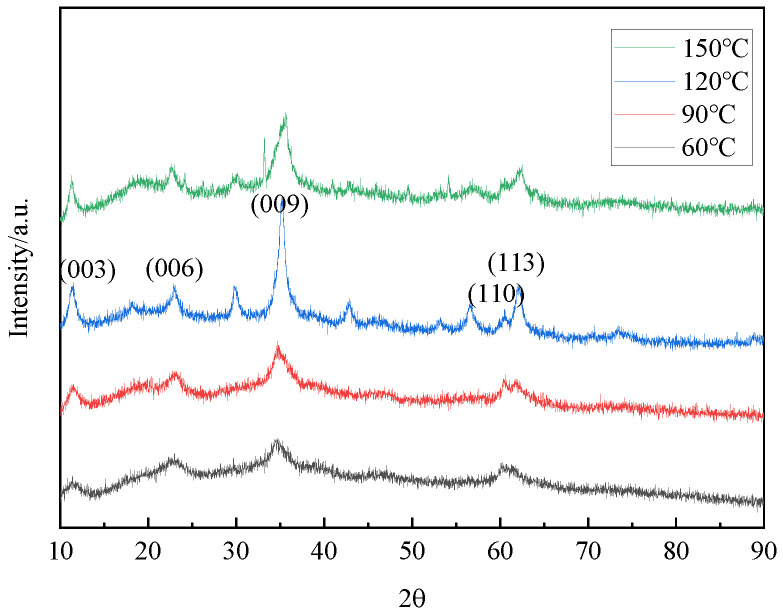
XRD patterns of hydrotalcite under different reaction temperature conditions.

**Figure 15:**
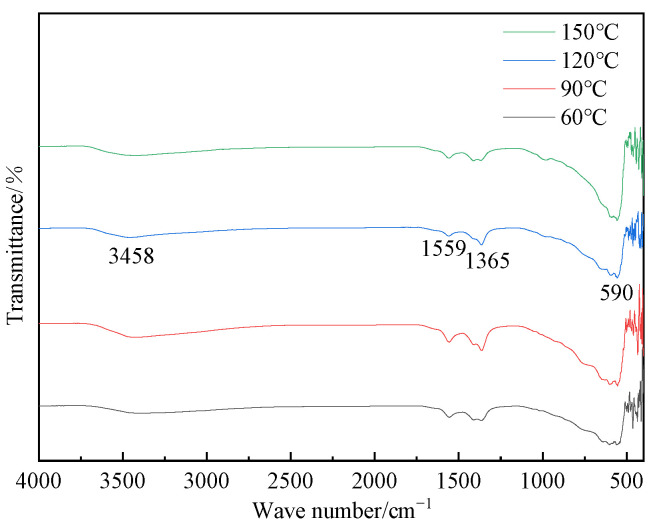
FT-IR plots of hydrotalcite under different reaction temperature conditions.

Figure 14 provided the XRD patterns of hydrotalcite under different reaction temperatures. By comparing the XRD patterns with those of standard magnesium–aluminum hydrotalcite, it could be seen that the diffraction peaks of the hydrothermal reaction products were the same as those of the standard magnesium–aluminum hydrotalcite when the temperatures are 60 °C, 90 °C, 120 °C, and 150 °C, proving that the hydrotalcite was generated in the sample. Furthermore, Figure 15 demonstrates corresponding peaks at 3458 cm^−1^, 1559 cm^−1^, 1365 cm^−1^, and 590 cm^−1^, providing additional supporting evidence for the synthesis of hydrotalcite.

In Table 3, the XRD data of the synthesized hydrotalcite at various temperatures are presented. The angles and d values of hydrotalcite synthesized under reaction system temperatures of 60 °C, 90 °C, 120 °C, and 150 °C are listed, along with the half-peak widths of hydrotalcite at (003), (006) and (009) crystal planes. It was observed that only the temperature at 90 °C and 120 °C satisfied the relationship of d_(003)_ = 2d_(006)_ = 3d_(009)_, indicating a more regular lamellar structure in the prepared hydrotalcite. The half-peak width of the hydrotalcite synthesized at 120 °C on the (003) crystal plane was 0.0119 rad smaller than that of 0.0124 rad at 90 °C. This suggested that the diffraction peaks on the (003) crystal plane of the hydrotalcite prepared at 120 °C were sharper and narrower, and exhibited improved crystallinity. Therefore, the optimum temperature for the preparation of hydrotalcite was 120 °C.

#### 3.2.3. The Effect of Reaction Time

**Figure 16:**
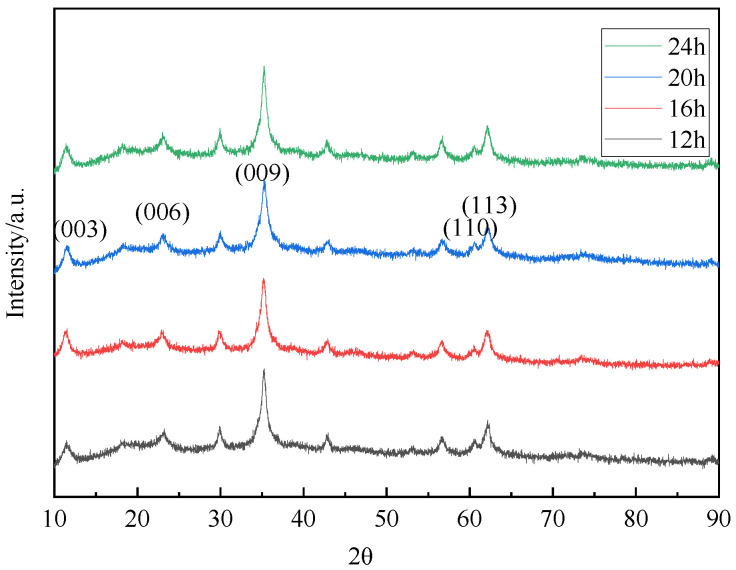
XRD pattern of hydrotalcite under different reaction time conditions.

**Figure 17:**
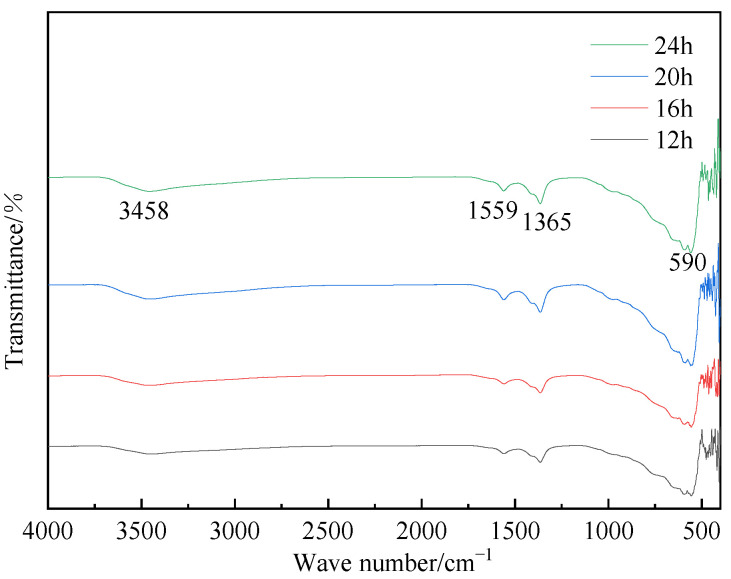
FT-IR plots of hydrotalcite under different reaction time conditions.

The XRD patterns of hydrotalcite under different reaction time conditions were shown in Figure 16. A comparison between these patterns and those of standard magnesium–aluminum hydrotalcite revealed that the diffraction peaks on the XRD patterns of the hydrothermal reaction products at 12 h, 16 h, 20 h, and 24 h aligned with those of the standard hydrotalcite. These peaks corresponded to the characteristic crystalline surfaces (003), (006), and (009), confirming the presence of hydrotalcite in the products. Further supporting evidence for the synthesis of hydrotalcite was provided by the FT-IR plots shown in Figure 17.

The XRD test data of hydrotalcite under different reaction time conditions were summarized in Table 4, which listed the angle and d value corresponding to the (003), (006), and (009) crystal planes, as well as the half-peak width on the (003) crystal plane of the samples synthesized at reaction times of 12 h, 16 h, 20 h, and 24 h, respectively. It could be observed that, at 12 h, 16 h, 20 h, and 24 h, the relationship of d_(003)_ = _(006)_ = 3d_(009)_ was satisfied, indicating a more regular lamellar structure. Notably, the half-peak width on the (003) crystalline surface was 0.0101 rad at a reaction time of 12 h, suggesting sharp and narrow diffraction peaks on this crystalline surface, indicative of better crystallinity. Combined with the above analysis and economic factors, 12 h was chosen for the subsequent process conditions.

The investigation of process conditions for the hydrothermal preparation of hydrotalcite involves three factors: pH, reaction temperature, and reaction time. The hydrotalcite could be successfully prepared when the pH of the solution system ranges from 10 to 12. However, at pH 9, the main products of the hydrothermal reaction were found to be CaCO_3_ and Fe_2_O_3_. The impact of the reaction temperature primarily influences the formation of the hydrotalcite phase in terms of its laminar structure and crystallinity. The effect of the reaction time was relatively small. The diffraction peaks of hydrotalcite generated at the reaction time of 12 h in the (003) crystal plane were sharp and narrow, and the crystallinity was better. In summary, the optimal process conditions for the hydrothermal preparation of hydrotalcite could be summarized as follows: a pH of 11, a reaction temperature of 120 °C, and a reaction time of 12 h.

### 3.3. Characterization of Steel-Slag-Based Hydrotalcite

#### 3.3.1. Morphology of Hydrotalcite

**Figure 18:**
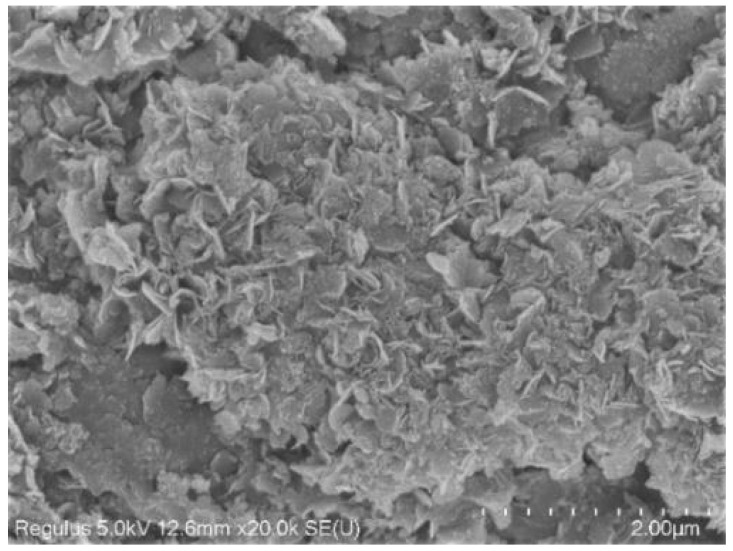
FESEM of hydrotalcite samples.

**Figure 19:**
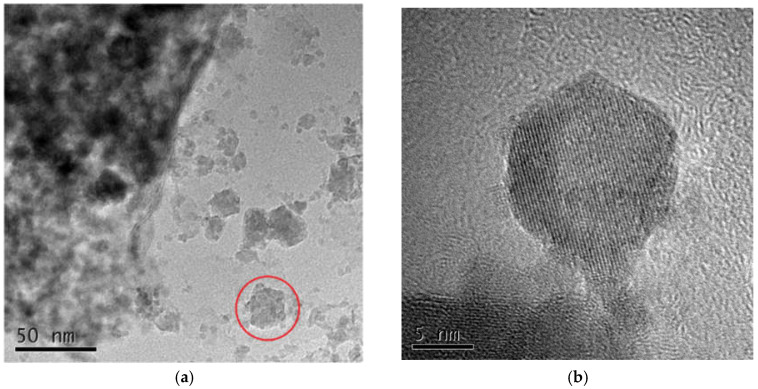
TEM of hydrotalcite samples. (**a**) TEM of the whole; (**b**) TEM at high resolution.

The FESEM images of the hydrotalcite sample were shown in Figure 18. The hydrotalcite had a hexagonal lamellar structure; the particles of the crystals were more uniformly dispersed, with better dispersion and a flat surface. Figure 19 displayed the TEM of the hydrotalcite sample images. Figure 19a provided an overall view, while Figure 19b displayed a high-resolution image. Figiure 19a depicted the hydrotalcite as having a flat, block-like shape composed of stacked layers, as indicated by the red circle. The high-resolution image reveals the presence of diffraction fringes within the hydrotalcite. The bright fringes correspond to elements with lower atomic numbers, emitted primarily by the anions in the interlayer. Conversely, the dark fringes represent elements with higher atomic numbers, emitted predominantly by the cations forming the lamellae. The orderly arrangement of atoms confirms the regular morphology of the synthesized hydrotalcite.

#### 3.3.2. Specific Surface Area Analysis of Hydrotalcite and Steel Slag

**Figure 20:**
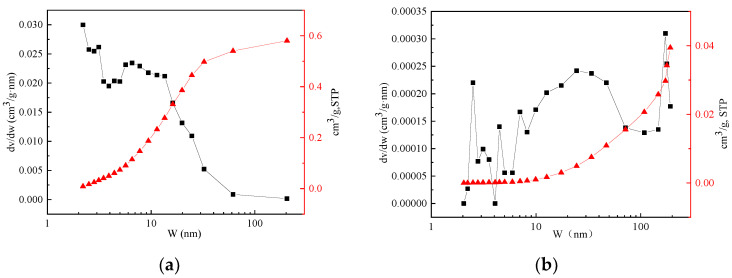
Specific surface area analysis of hydrotalcite and steel slag. (**a**) Hydrotalcite; (**b**) Steel slag.

Figure 20a displayed the specific surface area and pore size of hydrotalcite, while Figure 20b showed the specific surface area and pore size of steel slag. The data revealed that the pore size distribution of hydrotalcite was primarily concentrated within the range of 2–20 nm. Conversely, in the case of steel slag, the pore size distribution was more widely spread, spanning from 10–80 nm.

Table 5 presented the surface area, average pore size, and pore volume of hydrotalcite and steel slag. It demonstrated that hydrotalcite exhibits a specific surface area of 207.27 m^2^/g, an average pore diameter of 10.89 nm, and a pore volume of 0.56 cm^3^/g. In contrast, steel slag had a specific surface area of 3.88 m^2^/g, an average pore diameter of 40.68 nm, and a pore volume of 0.04 cm^3^/g. The pore diameter of hydrotalcite was approximately a quarter of that of steel slag, while its pore volume was significantly larger. Moreover, hydrotalcite possessed a substantially larger specific surface area compared to steel slag. A larger specific surface area typically corresponded to a more robust adsorption capacity. Accordingly, the larger specific surface area and pore volume of hydrotalcite made it a more favorable material for adsorption purposes when compared to steel slag.

### 3.4. Adsorption of Cl^−^ and SO_4_^2−^ from Salt-Washing Wastewater by Steel-Slag-Based Hydrotalcite

#### 3.4.1. Effect of Adsorption Time

**Figure 21:**
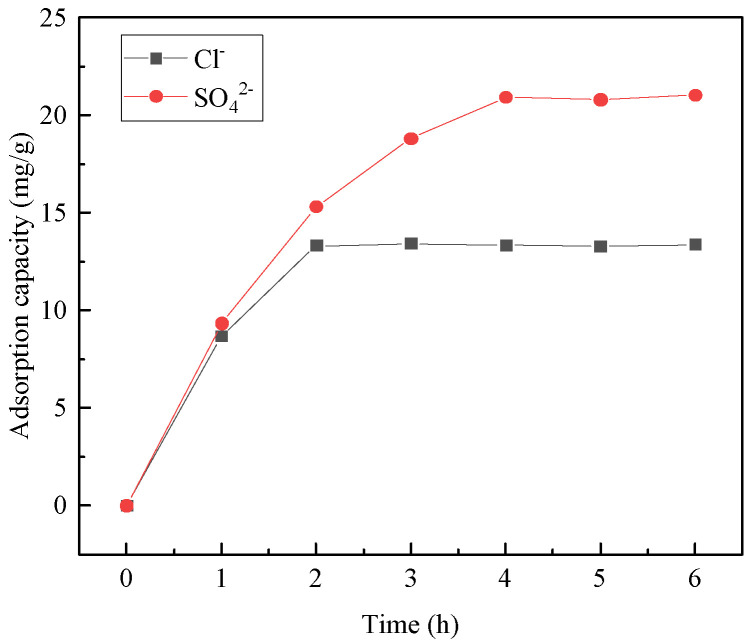
Adsorption of Cl^−^ and SO_4_^2−^ per unit mass of hydrotalcite.

**Figure 22:**
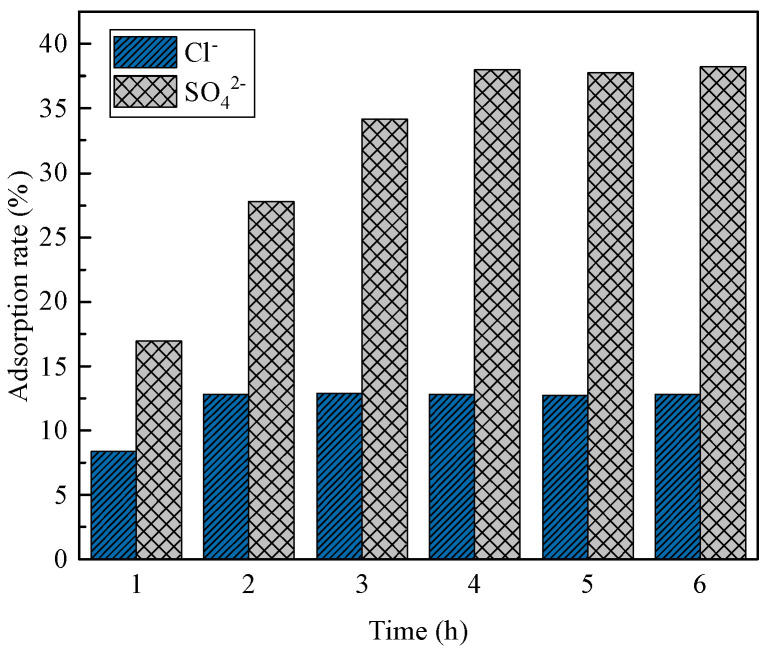
Removal rate of Cl^−^ and SO_4_^2−^ in salt-washing wastewater.

Figure 21 and Figure 22 illustrate the Cl^−^ and SO_4_^2−^ adsorption per unit mass of hydrotalcite and the corresponding removal rates from salt-washing wastewater. The figures demonstrate that, as the adsorption time increased within the first 2 h, both the Cl^−^ and SO_4_^2−^ adsorption per unit mass of hydrotalcite and their removal rates from the wastewater increased. Between 2 and 4 h, only the adsorption of SO_4_^2−^ per unit mass of hydrotalcite and the removal rate of SO_4_^2−^ from the wastewater continued to rise. After 4 h, the amount of Cl^−^ and SO_4_^2−^ adsorbed per unit mass of hydrotalcite, as well as the removal rates, reached a constant level. Consequently, the adsorption of hydrotalcite on salt-washing wastewater reached equilibrium at 4 h. At this stage, the amount of Cl^−^ adsorbed per unit mass of hydrotalcite was 13.3 mg/g, with a corresponding removal rate of 12.8% in the solution. Additionally, the amount of SO_4_^2−^ adsorbed per unit mass of hydrotalcite was 20.9 mg/g, with a removal rate of 38% in the solution. The adsorption capacity of SO_4_^2−^ was stronger than that of Cl^−^, possibly because of the higher charge density of sulfates, which resulted in a greater affinity for intercalation by sulfates [33].

#### 3.4.2. Multiple Adsorption of Steel-Slag-Based Hydrotalcite on Salt-Washing Wastewater

**Figure 23:**
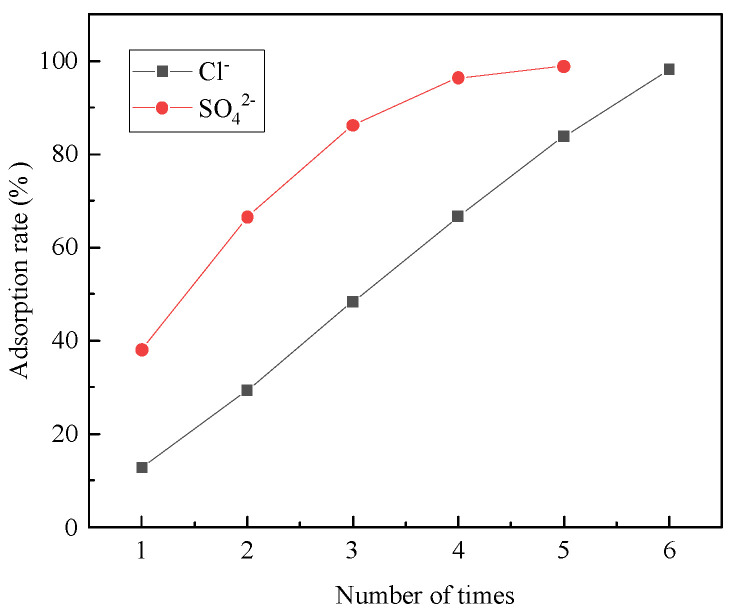
Removal rate of Cl^−^ and SO_4_^2−^ in salt-washing wastewater after the multiple adsorption.

Hydrotalcite was used to adsorb salt-washing wastewater multiple times, with subsequent measurements of Cl^−^ and SO_4_^2−^ concentrations in the wastewater. The removal rate of Cl^−^ and SO_4_^2−^ after repeated hydrotalcite adsorption was shown in Figure 23. It was evident from the figure that the amount of remaining Cl^−^ and SO_4_^2−^ in the wastewater had decreased with multiple adsorptions. The fourth adsorption achieved a 96.4% removal rate of SO_4_^2−^, while the sixth adsorption resulted in a 98% removal rate of Cl^−^, effectively treating Cl^−^ and SO_4_^2−^ in salt-washing wastewater. Consequently, hydrotalcite could partially remove Cl^−^ and SO_4_^2−^, offering a novel avenue for salt-washing wastewater treatment research.

## 4. Conclusions

The study aimed to examine the leaching mechanism of metal from steel slag using acetic acid as the reaction medium and determine the optimal leaching process for preparing hydrotalcite. Subsequently, hydrotalcite was synthesized through hydrothermal synthesis using the leach solution obtained from steel slag, and its structure was characterized. Additionally, the adsorption properties of hydrotalcite derived from steel slag on Cl^−^ and SO_4_^2−^ in salt-washing wastewater were investigated. The results are outlined below:

1. The acidolysis of steel slag exhibited the highest efficiency when conducted under specific conditions: an acetic acid concentration of 5 mol/L, liquid–solid ratio of 25:1, reaction temperature of 80 °C, and reaction time of 2 h. The ratio of trivalent metal cations to the total sum of divalent and trivalent metal cations in the leaching solution was determined to be 0.167. The relatively high concentration of metal cations in the leaching solution proved advantageous for the production of a greater quantity of hydrotalcite.
2. The optimum process conditions for the preparation of hydrotalcite by the hydrothermal method were pH 11, a reaction temperature of 120 °C, and reaction time of 12 h. FT-IR, FESEM, and TEM were used to analyze the hydrotalcite samples under the optimum conditions. The analytical results showed that the prepared hydrotalcite had a hexagonal lamellar structure, regular crystal structure, large specific surface area, and layered structure with ordered atomic arrangement.
3. In the adsorption experiments of steel-slag-based hydrotalcite in salt-washing wastewater, the adsorption amount of Cl^−^ per unit mass of hydrotalcite was 13.3 mg/g, and the removal rate of Cl^−^ in salt-washing wastewater reached 12.8%; the adsorption amount of SO_4_^2−^ per unit mass of hydrotalcite was 20.9 mg/g, and the removal rate of SO_4_^2−^ reached 38%. The adsorption of salt-washing wastewater was carried out several times, and it was found that the removal rate of SO_4_^2−^ reached 96.4% in the fourth adsorption, and the removal rate of Cl^−^ reached 98% in the sixth adsorption. These findings indicated that the adsorption process effectively eliminated Cl^−^ and SO_4_^2−^ from the salt-washing wastewater, essentially eliminating their presence.

## Figures and Tables

**Table 1:** Chemical composition of steel slag.

Component	LOI	SiO_2_	CaO	MgO	Al_2_O_3_	Fe_2_O_3_	SO_3_
wt%	2.81	16.86	39.11	8.45	6.38	19.73	0.48

**Table 2:** XRD test data of hydrotalcite under different pH conditions.

Sample	(003)			(006)		(009)	
	2θ_(003)_/°	d_(003)_/nm	β_(003)_/rad	2θ_(006)_/°	d_(006)_/nm	2θ_(009)_/°	d_(009)_/nm
pH = 10	11.58	0.7635	0.0142	23.42	0.3795	35.46	0.2529
pH = 11	11.48	0.7704	0.0119	23.24	0.3824	35.18	0.2549
pH = 12	11.24	0.7867	0.0094	22.78	0.3900	35.36	0.2537

**Table 3:** XRD test data of hydrotalcite under different reaction temperature conditions.

Sample	(003)			(006)		(009)	
	2θ_(003)_/°	d_(003)_/nm	β_(003)_/rad	2θ_(006)_/°	d_(006)_/nm	2θ_(009)_/°	d_(009)_/nm
60 °C	11.41	0.7751	0.0084	22.99	0.3865	34.40	0.2605
90 °C	11.32	0.7810	0.0124	22.72	0.3910	34.59	0.2591
120 °C	11.48	0.7704	0.0119	23.24	0.3824	35.18	0.2549
150 °C	11.30	0.7824	0.0102	22.66	0.3921	35.64	0.2517

**Table 4:** XRD test data of hydrotalcite under different reaction time.

Sample	(003)			(006)		(009)	
	2θ_(003)_/°	d_(003)_/nm	β_(003)_/rad	2θ_(006)_/°	d_(006)_/nm	2θ_(009)_/°	d_(009)_/nm
12 h	11.44	0.7727	0.0101	23.22	0.3827	35.26	0.2543
16 h	11.48	0.7704	0.0119	23.24	0.3824	35.18	0.2549
20 h	11.50	0.7688	0.0111	23.06	0.3854	35.24	0.2545
24 h	11.40	0.7756	0.0116	23.08	0.3850	35.24	0.2545

**Table 5:** Specific surface area data for hydrotalcite and steel slag.

Samples	Specific Surface Area (m^2^/g)	Average Pore Size (nm)	Pore Volume (cm^3^/g)
hydrotalcite	207.27	10.89	0.56
steel slag	3.88	40.68	0.04

## Data Availability

The data are contained within the article.
